# Survey data on employees’ perception of the impact of community development initiatives on the corporate image of oil and gas firms in Nigeria

**DOI:** 10.1016/j.dib.2018.06.077

**Published:** 2018-06-28

**Authors:** Bolanle Deborah Motilewa, Rowland E.K. Worlu, Chinonye Love Moses, Chinyerem Grace Adeniji, Gbenga Mayowa Agboola, Adeola I. Oyeyemi

**Affiliations:** Covenant University, Ota, Nigeria

**Keywords:** Community development, Corporate image, Social issues, Corporate Social Responsibility, Organisational performance, Nigeria

## Abstract

Several sources of today׳s pressure on managers in strategic decision-making are directly associated with social issues rather than traditional strategic management issues. It is believed that firms that invest in community development are more likely to operate in harmony in the society of their operations, as such reduce interference from their host community, thus leading to enhanced corporate image. Therefore, it becomes pertinent to present data to show the existence or otherwise of a relationship between community development initiatives and the firm׳s corporate image. This data is gotten from 336 respondents from four top oil and gas firms quoted in the Nigerian stock exchange. Responses wee gathered from the employees’ of the firms, as it is believed they have first hand information on the firm׳s corporate social responsibility policies. The data is purely descriptive and was gotten through quantitative methods, specifically through a survey questionnaire. The questionnaire had two sections; section A contained background questions, while section B consisted of questions that were specific to community development initiatives and corporate image. The Cronbach alpha internal consistency of the questionnaire revealed a reliability coefficient of 0.732, thus revealing a high consistency level. The field data set is made widely accessible to enable critical investigation into the subject.

**Specifications Table**

**Table:** 

**Subject area**	Business, Management
**More Specific Subject Area**	Corporate Social Responsibility, Community Development, Organisational Performance
**Type of Data**	Tables
**How Data was Acquired**	Customized researcher questionnaire
**Data Format**	Raw, analysed, Inferential Statistical data
**Experimental Factors**	The study population consists of the stakeholders of the top four firms listed in the oil and gas sector on the Nigerian stock exchange
**Experimental features**	The researcher-made questionnaire, which contained data on community development initiatives and corporate image were completed.
**Data source location**	South west Nigeria
**Data Accessibility**	Data is included in this article

**Value of data**

• The data presented seeks to show statistics on the firm׳s participation in community development initiatives and statistics on corporate image.
• The data carefully examines the employees’ perception of the firm׳s commitment to community development initiative, this gives an all rounded stakeholder view, unlike other data that do not consider the perception of the firm׳s employees.
• The data also portrays that the firm׳s participation in regular philanthropic activities such as infrastructure building, discourse on issues affecting host communities, providing access to credit, equity and basic banking products that otherwise may not be available to the community, promotion of poverty alleviation and sustainable development initiatives will give the society a perception that the corporation has it׳s interest at heart, thus leading to peace, harmony and closer ties between the corporation and its community.

## 1 Data

A total of three hundred and fifty copies of questionnaire were administered to respondents from the top four listed oil and gas firms in Nigeria׳s stock exchange. Table 1 below shows that 22.9% of the population of this study were from Firm 1, 27.3% from Firm 2, 27.8% from Firm 3 and 22% from Firm 4. This clearly shows that each firm for the study was well represented. The demographic characteristics of the respondents are also highlighted in Table 2 below.

**Table 1:** Sample frame for distribution of questionnaire.

**Name of firm**	**Number of employees**	**Percentage of total (%)**		**Questionnaire Distributed**
Firm 1	401	401/1748*100	22.9	80
Firm 2	477	477/1748*100	27.3	96
Firm 3	485	485/1748*100	27.8	97
Firm 4	385	385/1748*100	22.0	77
**Total**	**1748**		**100**	**350**

**Table 2:** Demographic characteristics of respondents.

**Demographic Characteristics**	**Items**	**Oil and Gas Firms**				**Total**
		**Firm 1**	**Firm 2**	**Firm 3**	**Firm 4**	
		**(%)**	**(%)**	**(%)**	**(%)**	
**Gender**	Male	38	40	59	57	194
		(49.4)	(44.4)	(62.8)	(76.0)	(57.7)
	Female	39	50	35	18	142
		(50.6)	(55.6)	(37.2)	(24.0)	(42.3)
**Total**		**77**	**90**	**94**	**75**	**336**
**Age**	Under 25 yrs	–	9	–	11	20
			(10.0)		(14.7)	(5.9)
	25–35 yrs	36	52	23	33	144
		(46.7)	(57.8)	(24.5)	(44.0)	(42.9)
	36–45 yrs	21	17	67	31	136
		(27.3)	(18.9)	(71.3)	(41.3)	(40.5)
	46 yrs +	20	12	4	–	36
		(26.0)	(13.3)	(4.3)		(10.7)
**Total**		**77**	**90**	**94**	**75**	**336**
**Length of Service**	Less than 5 yrs	57	51	70	22	200
		(74.0)	(56.7)	(74.5)	(29.3)	(59.5)
	5–10 years	20	21	20	26	87
		(26.0)	(23.3)	(21.3)	(34.7)	(25.9)
	11–15 years	–	4	4	25	33
			(4.4)	(4.3)	(33.3)	(9.8)
	16 yrs and above	–	14	–	2	16
			(15.6)		(2.7)	(4.8)
**Total**		**77**	**90**	**94**	**75**	**336**
**Status or Position**	Director	–	–	–	–	–
	Senior Manager	20	9	44	2	75
		(26.0)	(10.0)	(46.8)	(2.7)	(22.3)
	Analyst	–	28	17	23	68
			(31.1)	(18.1)	(30.7)	(20.2)
	Supervisor	57	8	2	41	108
		(74.0)	(8.9)	(2.1)	(54.7)	(32.3)
	Others	–	45	31	9	85
			(50.0)	(33.0)	(12.0)	(25.3)
**Total**		**77**	**90**	**94**	**75**	**336**
**Educational Status**	OND/NCE	–	13	–	7	20
			(14.4)		(9.3)	(5.9)
	HND/BSc	38	36	60	27	161
		(49.4)	(40.0)	(63.8)	(36.0)	(47.9)
	MSc/MBA/MEd	39	26	31	37	133
		(50.6)	(28.9)	(33.0)	(49.3)	(39.6)
	Others	–	15	3	4	22
			(16.7)	(3.2)	(5.3)	(6.6)
**Total**		**77**	**90**	**94**	**75**	**336**

**Statement of test statistics**

Given that the correlation co-efficient measures the degree to which two things vary together, this model correlated two variables: firm׳s participation in community development and corporate image in testing hypothesis two.

Table 3 showed the statistical significance of the two variables for each oil and gas firm: participation in community development and corporate image using the multiple regression. The statistics presented in the table above under R square is called the coefficient of determination and referred to as R^2^. The R Square tells how much of the variance in the dependent variable (corporate image) is explained by the independent variable (participation in community development). The F statistic tests the overall significance of the model. In this case, the value for each firm (Firm 1=.461, Firm 2=.629. Firm 3=.623 and Firm 4=.796) is expressed as a percentage, this means that the independent variable (participation in community development) explains Firm 1 (46.1%):, Firm 2 (62.9%), Firm 3 (62.3%) and Firm 4 (79.6%) of the variance in corporate image. Table 4.

**Table 3:** Model characteristics for each firm.

**Firm 1**			**Firm 2**			**Firm 3**			**Firm 4**		
**r**	**r**^**2**^	**Sig.**	**r**	**r**^**2**^	**Sig.**	**r**	**r**^**2**^	**Sig.**	**r**	**r**^**2**^	**Sig.**
.679^a^	.461	.000^b^	.793^a^	.629	.000^b^	.790^a^	.623	.000^b^	.892^a^	.796	.000^b^
F=64.167			F=149.024			F=152.278			F=285.311		

a Dependent Variable: Corporate_Image.

b Predictors: (Constant), Community_devept: Community development: regular philanthropic activities, discourse on issues affecting host community, promotion of poverty alleviation initiatives, promotion of sustainable development initiatives, financial support to host community.

**Table 4:** Model summary.

**Model Summary**			
Multiple R	R Square	Adjusted R Square	Apparent Prediction Error
.890	.792	.784	.208

^a^ Predictors: (Constant), Community development: regular philanthropic activities, discourse on issues affecting host community, promotion of poverty alleviation initiatives, promotion of sustainable development initiatives, financial support to host community.

^b^ Dependent variable: Corporate image.

The above Table 5 showed the statistical significance of the two variables of participation in community development and corporate image using the categorical regression.

**Table 5:** Model summary (ANOVA^a^).

**ANOVA**					
	Sum of Squares	df	Mean Square	F	Sig.
Regression	266.122	13	20.471	94.330	.000
Residual	69.878	322	.217		
Total	336.000	335			

^a^ Dependent Variable: Corporate_Image.

b Predictors: (Constant), Comm_Devept.

Table 6 shows the combined influence of the independent variables (regular philanthropic activities, discourse on issues affecting host community, promotion of poverty alleviation initiatives, promotion of sustainable development initiatives, financial support to host community) on corporate image (the dependent variable) of the firms. The result in Table 6 further establishes that the composite influence of firm׳s participation in community development did not occur by chance as it gives the F-ratio value of 94.330, which signifies the strength of the four independent variables (under participation in community development) as potent predictors of corporate image of the firms.

**Table 6:** Model summary (coefficients)^a^.

	Standardised Coefficients		df	F	Sig.
	Beta	Bootstrap (1000) Estimate of Std. Error			
The firm conducts community development activities on a regular basis	.144	.110	1	1.717	.191
The firm participates in local or regional committees to discus issues affecting its host community	.510	.143	4	12.758	.000
The firm gives regular financial support to its local community	.029	.076	2	.149	.862
The firm invests in activities that promote poverty alleviation in its host communities	.026	.114	2	.052	.949
The firm invests in activities that promote sustainable development in its host communities	.370	.128	4	8.385	.000

***Dependent Variable: Corporate_Image***.

The result in the Table 6 shows the staff opinion on firm׳s participation in community development to facilitate corporate image of the firms and it reveals that participation in local or regional committees to discus issues affecting its host community is a major predictor of corporate image which has the highest beta value of (*beta*=.510, *p*<.005, *Sig.* .000) than other variables: promotion of sustainable development initiatives scaled (*beta*=.370, *p<.005, Sig. .000*), regular community development (philanthropic) activities scaled (*beta*=.144, *p<.005, Sig. .004*). This means that participation in local or regional committees to discus issues affecting its host community makes the strongest unique contribution in influencing corporate image. While the financial support to host community and promotion of poverty alleviation initiatives are not statistically significant.

## 2 Experimental design, materials and methods

The data presented a quantitative research based on a descriptive research design to assess the effect of the firm׳s community development initiatives on its corporate image. Survey method was considered appropriate for data gathering.

The population of the study consists of the stakeholders of four (4) top oil and gas firms listed on the Nigerian stock exchange. The choice of these firms is in support of previous studies [1], [2], [3], [4] where it was statistically proffered that the study of corporate social responsibility is best situated in firms with top financial performance, as indicated by high stock price, which invariably means that the firm can carry out its economic obligations, and as such has resources to deal with social problems [5]. In total, there are one thousand, seven hundred and forty eight (1748) employees in all four firms. 350 employees were judiciously selected to partake in this research [6].

Data were collected from these organizations using an adapted researcher made questionnaire. A proportional analysis was conducted to determine the number of copies of the questionnaire to be distributed to the individual firms. The questionnaire is in two sections A and B. Section A contains background questions, section B consists of questions that are specific to the data provided, that is dimensions of firm׳s commitment to community development and corporate image, as it relates to information available to the employees.

The data was coded and keyed into the statistical package for social sciences (SPSS) version 22. Data was described using inferential statistical tests involving multiple regression analysis (Table 7).

**Table 7:** Total number of employees.

	**Firm 1**	**Firm 2**	**Firm 3**	**Firm 4**	**Total**
Number of Employees	401	477	485	385	1748

The researchers ascertained that respondents were well informed about the background and the purpose of the research. Every respondent was entitled to the opportunity to stay anonymous and their responses treated with utmost confidentiality. Permission was obtained from the appropriate authorities in the firms where copies of the research instruments were distributed.
