# Supplementary material for: Survey data on employees’ perception of the impact of community development initiatives on the corporate image of oil and gas firms in Nigeria
Source: Data Brief. 2018 Jun 28;19:1874–9. doi: 10.1016/j.dib.2018.06.077 (PMC6141378; doi:10.1016/j.dib.2018.06.077)
Supplement: Supplementary file 3 — Supplementary material [file mmc3.docx]

# RESEARCH QUESTIONNAIRE

Dear respondent,

This questionnaire is based on a study exploring the impact of community development on corporate image. I kindly ask for your voluntary co-operation in filling out this questionnaire.

Please note that the exercise is strictly for academic purpose and the information provided will be kept in strict confidence. Response to this questionnaire is absolutely voluntary and kindly note that you can decide at any point in time not to take part in this survey.

Thank you for your anticipated support and co-operation

**SECTION A: DEMOGRAPHIC DATA (Please tick whichever is applicable)**

1. Gender: Male Female
2. Marital status: Single Married Others
3. Age:
4. Under 25 years b. 25 – 35 years

c. 36 – 45 years d. 46 years and above

1. Length of service in the Oil and Gas Sector
2. Less than 5 years b. 5 – 10 years
3. 11 – 15 years d. 16 years and above
4. Position in the organisation
5. Director b. Senior Manager

c. Supervisor d. Analyst

d. Others, please specify ………………….

1. Educational qualification
2. OND/NCE b. HND/BSc.

c. MSc/MBA/M.Ed. d. Others

1. Additional Professional Qualifications:

Yes No

If Yes, Please specify ……………………………………………….

**SECTION B**

**Community Development and Corporate Image**

| **S/No** | **Item** | **SA** | **A** | **U** | **D** | **SD** |
| --- | --- | --- | --- | --- | --- | --- |
| 1 | Philanthropic activities are conducted on a regular basis |  |  |  |  |  |
| 2 | The firm participates in local or regional committees to discus issues affecting its host community |  |  |  |  |  |
| 3 | Regular financial support is given to the local community |  |  |  |  |  |
| 4 | The firm invests in activities that promote poverty alleviation in its host communities |  |  |  |  |  |
| 5 | The firm invests in activities that promote sustainable development in its host communities |  |  |  |  |  |
| 6 | The firm’s participation in philanthropic activities has led to reduced pressure from third parties |  |  |  |  |  |
| 7 | The firm’s participation in local committees to discus issues affecting its host community has strengthened its relationship with the government |  |  |  |  |  |
| 8 | The firm’s commitment to financially supporting the local community has strengthened its relationship with its host community |  |  |  |  |  |
| 9 | The firm’s investment in activities that promote poverty alleviation has helped to increase employee satisfaction in the firm |  |  |  |  |  |
| 10 | The firm’s investment in activities that promote sustainable development in its host communities has led to enhanced investors confidence |  |  |  |  |  |
